# The role of IL-6 in thyroid eye disease: an update on emerging treatments

**DOI:** 10.3389/fopht.2025.1544436

**Published:** 2025-04-14

**Authors:** Jennifer Murdock, John Nguyen, Brady J. Hurtgen, Cathy Andorfer, John Walsh, Andrea Lin, Christopher Tubbs, Kristine Erickson, Kimberly Cockerham

**Affiliations:** ^1^ Jennifer Murdock MD, PLLC, Miami, FL, United States; ^2^ School of Medicine, West Virginia University, Morgantown, WV, United States; ^3^ Tourmaline Bio, Inc., New York, NY, United States; ^4^ Department of Surgery, Sharp Grossmont Hospital for Neuroscience La Mesa, CA, United States

**Keywords:** IL-6, IL-6R, thyroid eye disease (TED), Graves’ ophthalmopathy, Graves’ orbitopathy, Graves’ disease, thyroid-associated orbitopathy (TAO)

## Abstract

Elevated serum interleukin-6 (IL-6) levels have been shown to correlate with disease activity in patients with thyroid eye disease (TED), a complex, heterogeneous, autoimmune disease affecting thousands of people worldwide. IL-6 plays a pivotal role in the pathogenesis of TED through three key mechanisms that together may contribute to inflammation, tissue expansion, remodeling, and fibrosis within the orbit. First, IL-6 drives an autoimmune response targeting the thyroid-stimulating hormone receptor (TSHR) by promoting the production of autoantibodies (i.e. TSHR-Ab, TSI), thereby triggering TSHR-dependent immune pathways. Second, IL-6 stimulates the activation and differentiation of orbital fibroblasts, which contributes to the inflammatory process and increase adipogenesis. Finally, IL-6 stimulates T-cell–mediated inflammation, amplifying the immune response within orbital tissues. Although corticosteroids and surgery have served as mainstays of TED treatment, a multimodal approach is often required due to the disease’s heterogeneous presentation and response to current treatment options. TED is a chronic, lifelong condition characterized by periods of exacerbation and remission, with inflammation playing a central role in disease progression and severity. Because inflammation can flare intermittently throughout a patient’s life, there is growing interest in targeting specific components of the immune system to reduce disease activity and severity. This review focuses on the current evidence supporting IL-6 as a key mediator of TED pathogenesis and explores its potential as a diagnostic biomarker and therapeutic target of the disease.

## 1 Introduction

Interleukin-6 (IL-6) is a multi-functional cytokine that regulates various physiological processes within multiple tissues. *IL-6* polymorphisms have been associated with varying risks for, or protection against several different diseases (1, 2). Multiple studies have reported a correlation between higher serum IL-6 levels and disease activity in patients with thyroid eye disease (TED) (3). TED is a complex autoimmune disease caused by local inflammation of the orbital tissues that can lead to visual dysfunction, vision loss, and facial disfigurement that alters the patient’s overall appearance and quality-of-life (QoL). The heterogenous presentation of TED requires a multimodal treatment approach that must also adapt over time to variable inflammatory activity. The therapeutic landscape changed with U.S. Food and Drug Administration (FDA) approval of Teprotumumab in February 2020. Since its introduction, other monoclonal antibodies are being evaluated for safety and efficacy in TED.

Targeted anti-IL-6 monoclonal antibody therapy has been used for TED patients in clinical studies (4). An understanding of IL-6 signaling is required to achieve a balance between targeting the pathological effects of IL-6-mediated inflammation and the unintentional concomitant abolition of its anti-inflammatory and pro-resolution effects. This review focuses on the current evidence suggesting a critical role for IL-6 as a key mediator of TED pathogenesis and discusses the potential utility of IL-6 as a biomarker and therapeutic target for TED.

## 2 Overview of IL-6

IL-6 is a pleiotropic cytokine produced by various lymphocytes and non-lymphocyte cells including T and B cells, fibroblasts, monocytes, mesangial cells, endothelial cells, keratinocytes, and tumor cells (5). Specifically, IL-6 is known to regulate the proliferation and differentiation of adult hematopoietic stem and progenitor cells in a paracrine manner (6). It induces several complement system proteins and the coagulation cascade, and also functions as an endogenous pyrogen to regulate thermogenesis (7). Notably, IL-6 plays a significant role in inflammation and immune responses by stimulating acute phase proteins (e.g., C-reactive protein [CRP], serum amyloid A, fibrinogen, and hepcidin), promoting T and B cell differentiation and maturation, and activating cells such as fibroblasts (8).

Additionally, IL-6 is a myokine, secreted from skeletal muscle during exercise and acting upon that muscle in paracrine and autocrine fashions. IL-6 also mediates anti-inflammatory and metabolic processes similar to an endocrine hormone. It has metabolic effects that allow for improved insulin signaling, enhanced insulin sensitivity, and increased fatty acid oxidation in skeletal muscles, and may also contribute to exercise-induced vascular remodeling (7). IL-6 is also pivotal for maintaining the integrity of the intestinal epithelium. Conversely, in inflammatory conditions, IL-6 harms this epithelial barrier by increasing intestinal permeability (9). Additionally, several chronic inflammatory conditions and different types of cancer feature aberrant hyperactivation of the IL−6/JAK/STAT3 signaling pathway (10).

IL-6 signals through three primary mechanisms: cis signaling, trans signaling, and trans-presentation (11). Briefly, cis signaling, otherwise termed classical signaling, involves binding of IL-6 to its membrane bound receptor (IL-6R), which activates intracellular pathways that influence local cell behavior (12). Conversely, trans signaling occurs when IL-6 binds to soluble IL-6R (sIL-6R), which can impact distant cells by binding to gp130 receptors (12). While trans-presentation, also known as cluster signaling, involves the presentation of IL-6-IL-6R complexes on the surface of one cell to another, contributing to downstream cell signaling and responses (11).

## 3 IL-6 and TED

### 3.1 Overview of TED

TED is a lifelong autoimmune disease with a prevalence of approximately five cases per 100,000 person-years (adjusted for age and sex) within the U.S (13). Although it can affect people of all ages, TED typically manifests during mid-life (e.g., 49 years of age), and occurs more frequently in women than men (14). Several factors have been associated with worsening severity of TED, including age, male sex, poorly controlled thyroid levels, radioactive iodine treatment, and smoking (15, 16). Many patients experience persistent or worsening orbital tissue inflammation for years, even after the initial presentation (e.g., 15.6% rate recurrence within 10 years from the initial episode) (17, 18). This local inflammation can lead to visual dysfunction, chronic pain, and facial disfigurement, resulting in characteristic changes to facial features and significant impacts on daily activities and functionality. Patients with TED ultimately experience a poorer QoL, social withdrawal, and mental health issues (15, 19–21). In two different studies evaluating the mental health of patients with TED, more than one-third of patients (36 - 42%) reported suffering from depression or anxiety, compared to one-fifth of the general U.S. adult population (17, 21).

Although the pathophysiology of TED is complex and not fully understood, the most widely accepted mechanism involves a loss of self-tolerance to the thyroid-stimulating hormone (TSH, or thyrotropin) receptor (TSHR) and insulin-like growth factor-1 receptor (IGF-1R) (22). These receptors are expressed together on fibroblasts in orbital and periocular tissues and interact with each other (23). This autoimmune response triggers an inflammatory cascade within the orbital, facial, and periocular tissues, leading to hypertrophy and fibrosis of the extraocular muscles, adipogenesis, eyelid retraction, and inflammation around the eyes (23, 24). Due to the presence of TSHR in orbital and periocular tissues as well as the thyroid, TED often occurs simultaneously with systemic Graves’ disease (GD), an autoimmune disorder affecting the thyroid gland that can lead to hyperthyroidism and abnormal thyroid hormone levels (25). However, in some cases, TED may present with normal euthyroid status or primary hypothyroidism (such as in Hashimoto’s thyroiditis) (26).

With persisting inflammation or flares of worsening inflammation, patients often experience symptoms such as proptosis, eyelid retraction, orbital pain, periocular redness and edema, and diplopia (26). As the tissues expand within the fixed, bony orbit, they can compress the optic nerve, leading to dysthyroid optic neuropathy and potential permanent vision loss (27, 28). Optic neuropathy in TED may also result from stretching of the optic nerve, which becomes more vulnerable to damage through circumferential straining (29). Inflammation, fibrosis, and hypertrophy of the extraocular fibers can lead to restrictive strabismus and diplopia significantly impairing patient functionality (23). Additionally as the globe moves anteriorly and the eyelids retract away from the eye, patients may suffer from ocular surface disease due to increased exposure of the ocular surface, altered blink dynamics, lagophthalmos, and evaporative dry eye disease (30).

### 3.2 Genetic associations in TED

*IL-6* polymorphisms have been associated with several diseases; though the directions of effect are variable (i.e., conferring increasing risk versus protective effect on a disease) (1, 2). Numerous studies have explored the genetic associations of IL-6 in GD and ocular diseases, including TED (31, 32). To date, four studies have specifically examined *IL-6* polymorphisms in TED (31, 33–35). The *IL-6*-174 G/C polymorphism (rs1800795) is of particular interest because it increases transcriptional activity of *IL-6* leading to higher downstream basal IL-6 levels (36). A meta-analysis of *IL-6*-174 G/C polymorphisms in ocular diseases also found a significant association in TED (31). However, when this polymorphism was assessed in 108 Polish-Caucasian patients with GD and clinically active TED, no association was found with serum IL-6 levels, thyroid autoantibodies, or the development and severity of TED (35).

### 3.3 The role of IL-6 in TED pathogenesis

**Figure 1:**
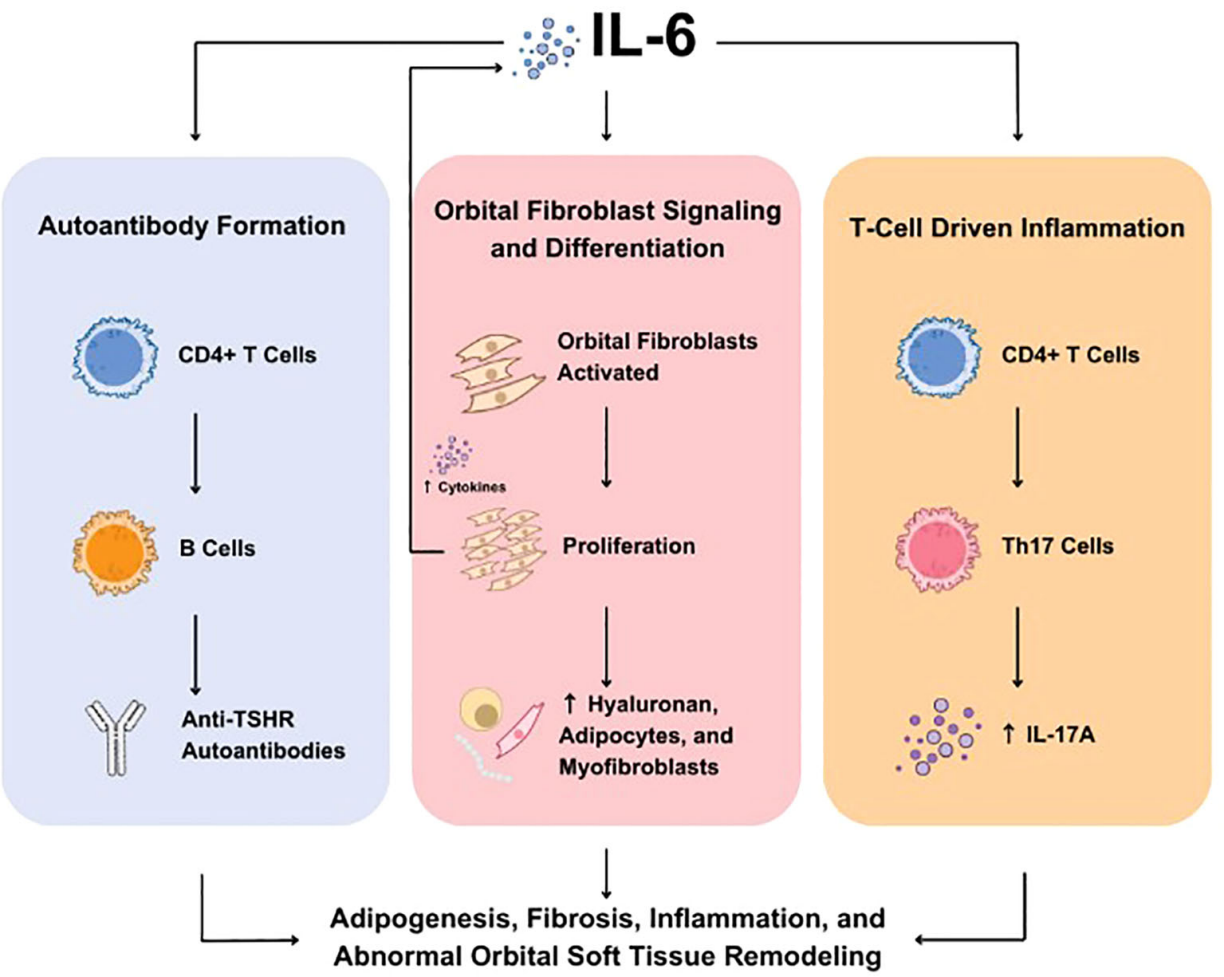
Role of IL-6 in TED. IL-6 is involved in three key molecular mechanisms underlying TED including autoantibody formation (left), OF signaling and differentiation (center), and T-cell driven inflammation (right). IL, interleukin; OF, orbital fibroblasts; TED, thyroid eye disease; Th, T helper; TSHR, thyroid-stimulating hormone receptor.

Production of IL-6 is regulated by several cell types involved in TED, including fibrocytes, orbital fibroblasts (OFs), adipocytes, and orbital macrophages, and is also the result of OF-lymphocyte crosstalk (37–41). Studies suggest that IL-6 drives TED pathogenesis through three key mechanisms that together may contribute to inflammation, tissue expansion, remodeling, and fibrosis within the orbit. First, IL-6 drives an autoimmune response targeting the TSHR by promoting the production of autoantibodies (i.e. TSHR-Ab, TSI), thereby triggering TSHR-dependent immune pathways. Second, IL-6 stimulates the activation and differentiation of OFs, which contributes to the inflammatory process and increases adipogenesis. Finally, IL-6 stimulates T-cell–mediated inflammation, amplifying the immune response within orbital tissues (**Figure 1**) (23).

### 3.4 Autoantibody formation

The pathophysiology of TED appears to involve autoantibody activation of TSH and IGF-1 receptors, both of which are overexpressed in orbital tissue and on OFs of patients with TED. Serum levels of TSHR antibodies (TSHR-Abs) correlate positively with TED severity (42, 43). Specifically, thyroid-stimulating immunoglobulin (TSI) is predictive of TED and correlates with both the activity and severity of the disease (43). TSHR-Abs promote cellular activation, inflammation, early adipocyte differentiation and stimulation of hyaluronan and group-specific antigen (GAG) production which all lead to orbital tissue changes and remodeling (42). In severe cases, these processes can lead to vision loss (44–46).

IL-6 plays a supporting role in autoantibody formation, as well as in the maturation of memory B cells and plasma cells (47). IL-6 also promotes the generation of T follicular helper (Tfh) cells, which are necessary for germinal center formation, antibody affinity maturation, and the development of high-affinity antibodies (potentially including TSHR-Ab and TSI) (48, 49). Data on Tfh cells in TED are limited, but studies show that Tfh cells in peripheral blood mononuclear cells from TED patients are significantly elevated (10.83%) compared to non-TED controls (0.36%) (50). While no correlation was found between Tfh cell levels and the Clinical Activity Score (CAS), a positive correlation was observed with TSI levels (50). Additionally, Tfh cells were found to be increased locally in orbital tissue in patients with dysthyroid optic neuropathy compared to those with inactive TED (51). Overall, autoantibodies play a central role in TED pathogenesis and disease presentation.

### 3.5 Orbital fibroblast signaling and differentiation

OFs are key to the pathogenesis of TED and contribute to soft tissue inflammation and hypertrophy (24, 52). In early active TED, the extraocular muscles, as well as adipose and connective tissues, become infiltrated by mononuclear cells (including CD4+ and CD8+ T cells, monocytes, macrophages, B cells, plasma cells, and fibrocytes), which activate OFs through direct cellular interactions or inflammatory mediators, including IL-6 (53).

Furthermore, OFs may be primed to be hyper-responsive to IL-6. Following activation by IL-1β, OFs produce significantly higher levels of IL-6 than dermal fibroblasts (54). Basal levels of IL-6R are also notably higher in OFs compared to dermal fibroblasts (54). This site-specific over-expression of these molecules may offer potential therapeutic targets for TED. Additionally, the IL-6 pathway can influence OFs in TED by modulating several effector functions and molecules that play important roles in the disease. For instance, the proinflammatory phenotype of OFs in TED is enhanced following stimulation with IL-6 and sIL-6R, leading to upregulation of nuclear factor kappa B, chemokines (such as monocyte chemoattractant protein-1 [MPC-1]), molecules involved in antigen presentation (e.g., major histocompatibility complex-I [MHC-I]), and co-stimulatory molecules (e.g., CD40 and intercellular adhesion molecule-1 [ICAM-1]) (39).

TSHR stimulation on OFs has also been implicated in the pathogenesis of TED (55). IL-6 has been shown to upregulate TSHR gene expression in OFs. Cultured OFs isolated from patients with severe TED displayed TSH-dependent cAMP production and increased TSHR mRNA following IL-6 stimulation, whereas OFs from individuals without GD or TED did not (45). However, another study found that stimulating CD34+ and CD34- fibroblasts with IL-6 or IL-6 plus sIL-6R did not significantly increase TSHR expression (39, 45). Although IL-6 may influence autoantigen levels in OFs, further research is needed to clarify this relationship.

IL-6 has also been implicated in fibrotic diseases, including ocular fibrosis (56). IL-6, in combination with sIL-6R, has been shown to upregulate expression of extracellular matrix proteins—such as fibronectin, collagen I, alpha-smooth muscle actin (α-SMA), and tissue inhibitor of metalloprotease 1 (TIMP-1)—in OFs (56). These *in vitro* effects were attenuated by the addition of tocilizumab, a recombinant humanized monoclonal anti-IL-6R antibody (39). While it’s exact role in TED fibrosis remains unclear, these findings suggest potential involvement of IL-6.

Although limited, some evidence suggests that IL-6 may play a role in driving adipogenesis in OFs derived from TED patients (45). Stimulation of adipogenesis is associated with increased TSHR expression and TSH-dependent cyclic adenosine monophosphate (cAMP) production. The addition of IL-6 during adipocyte differentiation resulted in enhanced expression of both, indicating a potential influence on adipogenesis (45). However, further studies are needed to confirm these findings.

OFs also produce IL-6 in response to various stimuli in TED. Several studies suggest that IL-6 is produced by CD34+ OFs in a TSHR-dependent manner, with TSH and M22 (an activating anti-TSHR mAb) driving IL-6 production (37). Several studies suggest that IL-6 is produced by CD34+ OFs in a TSHR-dependent manner. This production is driven by TSH and M22, an activating anti-TSHR monoclonal antibody (45).

IL-6 production by OFs can also occur through direct cellular costimulation and cross talk. OFs express CD40, a costimulatory molecule involved in cellular activation (38). The interaction between CD40 on OFs and membrane-bound CD40 ligand (CD154) results in the production of inflammatory mediators, including IL-6 (38, 57). This effect was observed in OFs from patients with stable TED but not in control OFs (38). IL-6 is also produced through cross talk between OFs and B and T cells (40, 41). Whether this is dependent on direct costimulation or indirect cross talk remains unclear.

Additionally, OF production of IL-6 is influenced by soluble mediators, including prostaglandin E2 (PGE2) and interleukin-1 beta (IL-1β) (54, 58). PGE2, produced by OFs, stimulates a dose-dependent production of IL-6 (58). Similarly, IL-1β stimulation results in time- and concentration-dependent IL-6 production by OFs (54). Both studies reported greater IL-6 production in PGE2- and IL-1β-stimulated OFs compared to dermal fibroblasts.

In summary, OFs in TED are hyperresponsive to IL-6, which promotes inflammatory and fibrotic responses that likely contribute to the pathogenesis of TED. These findings suggest that IL-6 and its signaling pathways can serve as potential therapeutic targets in the treatment of TED.

### 3.6 T-cell-driven inflammation and fibrosis

In conjunction with TGF-β, IL-6 is essential for the differentiation of naïve CD4+ T cells into the T helper-17 (Th17) subset, which promotes inflammatory responses and modulates fibrosis, in part through the secretion of the cytokine IL-17A (59, 60).

Pathogenic Th17 cells are upregulated in circulation and are found to be a dominant T helper cell type within the orbit of patients with TED (41, 61, 62). Both circulating and orbital Th17 cells positively correlate with the CAS, and orbital Th17 cells negatively correlate with visual acuity (41, 62). In cases of active, severe TED with dysthyroid optic neuropathy, Th17 cells adopt a Th17.1 phenotype (or Th1-like Th17), where they coexpress the master transcription factors T-Bet and RORγt and produce both IFN-γ and IL-17A (both Th1 and Th17, respectively (63). Th17.1 cells appear to be more pathogenic than Th17 cells, displaying enhanced proliferation, increased production of proinflammatory cytokines, and reduced suppression by regulatory T cells (64). Interestingly, it is hypothesized that inhibiting the IL-6 pathway may prevent Th17.1 polarization, providing a mechanistic explanation for reports of tocilizumab use in steroid-resistant, severe TED (65).

The Th17 cell population is thought to influence OF inflammatory status, fibrosis, and adipogenesis in TED (60). Coculture of Th17 cells or IL-17A with OFs results in robust production of IL-6 along with IL-8 and monocyte chemoattractant protein-1 (MCP-1) (41). IL-17A impacts fibrosis in OFs by enhancing TGF-β-induced extracellular matrix production—such as collagen I, fibronectin, matrix metalloprotease-2 (MMP-2), and TIMP-1—in TED-derived OFs (41, 61). The same study reported that IL-17A synergizes with TGF-β to drive enhanced myofibroblast differentiation in CD90+ OFs. The role of IL-17A in adipogenesis remains unclear, as studies suggest both inhibitory and stimulatory effects on adipogenesis in OFs (41, 66).

Similar to the role of IL-6 in T follicular helper cell development for autoantibody production, IL-6 is required for Th17 differentiation and the downstream effector functions that drive inflammation and fibrosis in TED. It remains to be determined how IL-6 blockade might impact these mechanisms in TED.

## 4 Treatments for TED

The goal of TED treatment is essentially to prevent the worsening of symptom severity and reversal of sequelae of inflammation. Therefore, early intervention with durable treatment options that reduce inflammation is of the utmost critical importance. However, treatment of TED is challenging due to the potential need for multimodal and long-lasting therapeutic strategies (15). Corticosteroids and surgery have been the mainstay of treatment of TED for decades, but these approaches do not fully address the heterogeneity of disease presentation. Furthermore, although teprotumumab has been approved by the FDA, its access is limited outside the U.S.

Patients with mild symptoms that do not significantly affect their QoL are often provided with supportive care (including vitamin D and selenium supplementation, artificial tears without preservatives, and optimizing thyroid function). For patients whose symptoms affect their everyday functioning including their ability to work, read, drive, or interfere with their professional and personal interactions, corticosteroids or teprotumumab are typically used (67, 68). Other options include orbital irradiation, steroid-sparing agents, and biologics (**Table 1**) (28, 70, 71). Notably, many existing non-surgical treatments aim to reduce inflammation but do not address all symptoms of TED (i.e., eyelid retraction, diplopia). Despite good results with current treatment options, TED patients still may have residual proptosis, eyelid retraction, or diplopia, thus requiring surgical intervention. Nonetheless, these symptoms can also recur or worsen with time, necessitating multiple rounds of treatment protocols or corrective surgeries. Ultimately, TED regression and recurrence continue to be a challenge (19, 72, 73).

**Table 1:** Summary of treatment options for patients with TED.

Treatment	Description
Steroids	Usually given as a first-line treatment, oral corticosteroid and intravenous glucocorticoid therapy must be monitored carefully due to their large side effect profile (28)
Surgery	Orbital decompression, strabismus surgery, and eyelid procedures (28) as well as corrective procedures to address symptoms affecting QoL of patients with inactive and mild disease (19)
Radiation therapy	Orbital radiation therapy can help minimize the dose and duration of corticosteroid therapy (69)
Supportive care	Managing symptoms with over-the-counter remedies (e.g., lubricating eye drops, ointments) and vitamin D and selenium supplementation as well as lifestyle management (e.g., minimizing smoking) (28, 70)
Watchful waiting	For patients whose disease is not progressive and who are not severely symptomatic, watchful monitoring is an option, as the disease will remit partially or completely throughout its lifecycle (19)
Steroid sparing agents and biologics	Monoclonal antibodies or biologics such as rituximab, teprotumumab, and tocilizumab may be used to minimize inflammation in TED patients (28)

TED, thyroid eye disease; QoL, quality of life.

### 4.1 Immune-targeting and inflammation-targeting treatments

Given TED is a progressively worsening disease generally characterized by an initial inflammatory phase, there is growing interest in therapeutically targeting specific components within the immune system to reduce the magnitude of disease. Targeted biologics, including monoclonal antibodies, have been an established therapeutic strategy in other autoimmune and inflammatory conditions and are now being further explored as steroid-sparing agents for the treatment of TED. Currently in use or under investigation are antibodies targeting CD20, IL-6, IL-6R, IL-11R, IGF-1R, and the neonatal Fc receptor (FcRn). The monoclonal antibodies teprotumumab (anti-IGF−1R; FDA-approved), rituximab (anti-CD20; off-label), and tocilizumab (anti-IL-6R; off-label) have been evaluated for TED treatment in clinical trials (**Table 2**) (28). Interestingly, a meta-analysis of 12 trials (five randomized controlled trials [RCTs] and seven observational studies) comparing rituximab, teprotumumab, or tocilizumab in 448 patients with active and moderate-to-severe TED, found that tocilizumab treatment resulted in a greater treatment response (CAS reduction, efficacy response rate, disease inactivation rate), reduction in proptosis, and the lowest association for adverse events (based on odds ratio of the three treatments, while diplopia improved the most with teprotumumab treatment (75).

**Table 2:** Summary and comparison of characteristics of biologic treatments for TED (74)..

Therapy	FDA approval for TED	Administration	Efficacy	Safety
Teprotumumab (anti-IGF-1R)	Approved (2020)	Intravenous	Reduced proptosis (79%-83%) Improved CAS (69%) Reduced diplopia (68%)	Hearing loss (10%) Hyperglycemia (10%) Muscle spasms (25%) Alopecia (13%)
Tocilizumab (anti-IL−6R)	Not approved	Intravenous, subcutaneous	Improvement of CAS by ≥ 2 points overall (93%) Mean proptosis reduction of 1.5 mm No change in diplopia	Hypercholesterolemia (10%) Neutropenia (10%) Elevated liver enzymes (5%) Infusion reactions (7%-8%)
Rituximab (anti-CD20)	Not approved	Intravenous	Mixed results on improvement of CAS, proptosis, and motility	Infusion reactions (23%-32%) Rash (15%) Angioedema (11%) Infections (serious, 5%; overall, 31%) Hepatitis B reactivation (rare) Progressive multifocal leukoencephalopathy (rare) Cytopenias (lymphopenia, 40%; neutropenia, 6%; anemia, 3%; thrombocytopenia, 2%) Cardiac events (1%-2%) Hyperglycemia (9%)

CAS, clinical activity score; FDA, Food and Drug Administration; IGF-1R, insulin-like growth factor 1 receptor; IL, interleukin; R, receptor; TED, thyroid eye disease.

### 4.2 IL-6 pathway inhibition overview

The pleotropic and proinflammatory effects of IL-6 are well recognized. Targeting the IL-6 pathway and related-immune processes early in this progressive disease may reduce the duration of symptoms, limit the magnitude of the disease, address the physical changes that can potentially persist, and even allow for long-term disease inactivation. Since 2014, numerous reports have been published surrounding therapeutic targeting of IL-6 signaling in over 200 patients with TED; these have been previously reviewed elsewhere (76). Overall, these studies reported reductions in CAS, proptosis, and thyroid autoantibodies (76). However, reductions in diplopia after treatment with IL-6 antibodies are inconsistent and contain several study limitations and research gaps discussed later in this manuscript. Even though a more comprehensive review has been published previously, it is important to briefly review what is currently known about IL-6 and the emerging data coming down the pipeline (76).

### 4.3 Targeting IL-6R: tocilizumab and sarilumab

Tocilizumab is a recombinant humanized monoclonal anti-IL-6R antibody with an excellent safety profile even in vulnerable populations like the elderly (77, 78). The majority of published studies targeting the IL-6 pathway in TED involve intravenous use of tocilizumab. Duarte et al. conducted a systematic review which suggested tocilizumab to be mostly effective in reducing inflammatory signs during active TED, with an improvement of ≥ 3 points CAS, and an overall relapse rate of 8.2% (76). In numerous studies tocilizumab also resulted in marked reductions in proptosis (76). A meta-analysis of 12 studies (N=219) including patients with active, steroid-resistant TED who were treated with intravenous tocilizumab reported similar findings: CAS reduction of 4.6 points, proptosis reduction of 2.04 mm, diplopia response rate of 48%, and a reactivation rate of 1% (4).

There is currently only one RCT with published data: a Phase 3 study that enrolled 32 patients with moderate-to-severe corticosteroid-resistant TED (79). Patients received tocilizumab (8 mg/kg at weeks 0, 4, 9, and 12) or placebo. At 16 and 40 weeks from baseline, respectively, 14/15 (93%) and 13/15 (87%) tocilizumab-treated patients had improved CAS (≥2 points) compared with 10/17 (59%) placebo-treated patients (for both timepoints) (79). Although there was a significant reduction in proptosis at week 16, this result did not remain at week 40; at both time points the median change was –1.5mm (79). No patients withdrew from the study due to an adverse event (79). Only two tocilizumab-treated patients experienced a serious adverse event; though it is unclear whether this was due to the study drug (79). One patient who was treated with hydrazides for latent tuberculosis, experienced a moderate transaminase elevation at week 8 that normalized after discontinuing hydrazides, while another patient experienced acute pyelonephritis at week 30 (79). Studies have also reported reductions in antithyroid autoantibodies following treatment with tocilizumab in TED. In one of the first meta-analyses evaluating changes in thyroid autoantibodies following treatment with tocilizumab, TSHR-Ab levels were reported to be reduced from baseline following tocilizumab treatment in TED in five studies (10.62-IU mean reduction in TSHR-Ab) (4). A cohort study of nine patients with active TED who received tocilizumab following intolerance to or disease progression following systemic corticosteroid treatment found similar outcomes (74). After an average of 4.2 tocilizumab infusions, patients’ CAS, TED Scale score, and TSI levels were reduced to 0.4 ± 0.5 (reduction of 6.3 points), 1.2 ± 1.1 (reduction of 9.0 points), and 201% (reduction of 240%), respectively (80). After an average follow-up of 24 months post treatment, no patients experienced recurrence of active disease (80). The findings from these studies are noteworthy given the role of IL-6 in B cell and antibody-mediated immune responses, as well as the role of TSI in TED.

In addition to steroid-resistant patients, tocilizumab use has also been described in patients with moderate-to-severe, longstanding TED resistant to treatment with: corticosteroids, methotrexate, radiation, and surgery. Patients (N=9) were followed for an average of 24.6 weeks. Positive clinical responses were observed in all patients including improvements in proptosis, eyelid edema, diplopia, extraocular movement, visual acuity, color vision, and TSI levels (74). Investigators documented an overall decrease in CAS indicating improved overall patient burden (74). These studies support the potential clinical utility of the IL-6 pathway inhibition for the treatment of recalcitrant TED.

While the studies reporting efficacious results for tocilizumab in TED have provided a basis for hypotheses surrounding IL-6 pathway inhibition, these studies contain several pitfalls that may reduce their strength and limit their interpretation. To date, there is limited data from randomized, placebo-controlled clinical trials. In fact, the lack of controlled trials even prevented a Cochrane database review of tocilizumab in TED as no studies met the standard for review and inclusion (81). Another limitation is the lack of a treatment-naïve population; most studies have evaluated tocilizumab in a steroid-failure population. Therefore, the effect of targeting the IL-6 pathway early in the course of active disease in a treatment-naïve population is unknown. Further research, particularly in the form of RCTs, is required to fully understand tocilizumab’s impact, appropriate dosage, and administration in patients with TED.

Sarilumab is another anti-IL-6R Ab with reported use in TED, but has not yet been studied in an RCT setting (82). Sarilumab is a fully human monoclonal anti-IL-6R Ab that blocks both cis and trans IL-6 signaling as well as IL-6 trans presentation (or cluster signaling) (83, 84). It was first approved by the FDA in 2017 for the treatment of adults with moderate-to-severe active rheumatoid arthritis (RA) who have had an inadequate response or intolerance to one or more disease-modifying antirheumatic drugs. Sarilumab is also approved for RA in the European Union and Japan (85). An observational case series abstract describes the use of sarilumab (200 mg, subcutaneous, every 15 days for a mean of 11 months) in active, moderate-to-severe TED in five patients. In this case series, CAS was reduced by an average of 3 points following treatment with sarilumab; all patients achieved TED inactivity and reduced disease severity, along with reductions in antithyroid antibody levels (82).

### 4.4 Targeting IL-6: sirukumab, siltuximab, and olamkicept

Sirukumab, siltuximab, and olamkicept are human monoclonal anti-IL-6 Abs that block classic and trans IL-6 signaling (**Table 3**) (84, 86, 87). Siltuximab received FDA approval in 2014 for patients with multicentric Castleman’s disease who are HIV negative and HHV-8 negative (88). While sirukumab demonstrated efficacy for the treatment of RA in a Phase 3 trial (89). Further, olamkicept is a soluble gp130-Fc-fusion-protein currently in development that inhibits trans IL-6 signaling, affecting IL-6-driven chronic inflammation (90). Currently, there are no RCTs investigating the use of these monoclonal antibodies in TED.

**Table 3:** Treatments that target IL-6 signaling with no current RCTs for TED.

Interventions	Status	Disease State	Administration
Sarilumab (anti-IL-6R)	Approved (2017, 2023, 2024)	RA, PMR, pJIA	Subcutaneous
Sirukumab (anti-IL-6)	Completed Phase 3 (NCT01604343)	RA	Subcutaneous
Siltuximab (anti-IL-6)	Approved (2014)	Castleman’s disease	Intravenous
Olamkicept (anti-IL‐6/sIL‐6R)	Completed Phase 2 (NCT06298032)	UC	Intravenous

IL, interleukin; pJIA, polyarticular juvenile idiopathic arthritis; PMR, polymyalgia rheumatica; RA, rheumatoid arthritis; R, receptor; s, soluble; TED, thyroid eye disease; UC, ulcerative colitis.

Based on data from two small, single-arm, single-dose studies comparing the immunological effects of siltuximab (which targets IL-6) and tocilizumab (which targets IL-6R), several differences were observed. Firstly, inhibition of IL-6R but not IL-6, led to persistent suppression of IL-6–induced phosphorylated-signal transducer and activator of transcription 3 (p-STAT3) and declines in inducible T-cell costimulator expression on T follicular helper cells (91). While IL-6 inhibition reversed T effector resistance to Treg-mediated suppression and enhanced T cell production of regulatory cytokines (91). In addition, IL-6 and IL-6R inhibition had opposing effects on T-cell receptor-induced p-STAT3 signaling (91). However, a recent meta-analysis of six different RCTs revealed comparable efficacy and safety between IL-6 and IL-6R inhibitors, thus suggesting that there may not be a meaningful clinical difference between targeting IL-6 versus IL-6R (92).

The clinical implications of these observations are currently unknown. Although the protective and reparative functions of IL-6 in TED are currently poorly understood and underappreciated, it will be important to understand how these functions may be impacted by inhibition of IL-6R (trans- and classical-signaling) versus IL-6. A theoretical advantage of targeting IL-6 versus IL-6R, is that IL-6 is found at low levels in circulation compared with IL-6R, which may impact drug load, half-life, and effectiveness (93).

Controlled clinical studies are underway that may shed light on these considerations. There are three Phase 2/3 RCTs are currently recruiting (as of October 2024) to evaluate anti-IL-6 (pacibekitug) or -IL-6R antibodies (satralizumab) as potential treatments for TED (**Table 4**).

**Table 4:** Ongoing and upcoming Phase 2 or 3 trials evaluating treatments for TED that target IL-6 or IL−6R.

Phase	Interventions	Study	Trial ID	N	Primary completion date (estimated)
Phase 2	Pacibekitug (anti-IL-6)	spiriTED	NCT06088979 (94)	81	Nov 2025
Phase 3	Satralizumab (anti-IL-6R)	SatraGO-1	NCT05987423 (95)	131	Sep 2025
Phase 3	Satralizumab (anti-IL-6R)	SatraGO-2	NCT06106828 (96)	127	May 2025

IL, interleukin; R, receptor; TED, thyroid eye disease.

## 5 Potential utility of IL-6 as a biomarker for TED

There has been a strong interest to identify molecular biomarkers that provide clinical utility in diagnosis, management, and treatment of TED, since there are currently no acceptable options (3, 97, 98). Studies evaluating IL-6, sIL-6R, and surrogates of IL-6 pathway activation in those with TED suggest the potential utility of IL−6 pathway activation as a biomarker.

IL-6 levels have been evaluated in both serum and tears of those with TED. Numerous studies have reported significant increases of IL-6 levels within tears isolated from those with TED compared with controls. Tear-derived IL-6 was found to positively correlate with CAS, disease activity, and lacrimal gland enlargement; however differences were not observed between TED and GD (99–103). IL-6 levels in tears were also found to decrease with CAS reductions following treatment of active, moderate-to-severe TED with intravenous methylprednisolone (103). Additionally, a few studies have evaluated levels of IL-6 in the serum of patients with TED. In a study comparing serum levels in patients who had GD with (n=47) or without (n=29) ophthalmopathy, IL-6 levels were significantly elevated in those displaying ocular disease (104); however data correlating serum IL-6 levels with inflammation and activity are inconsistent (104, 105).

IL-6R can be generated by alternative splicing or cleavage from the cell membrane and enter circulation as sIL-6R, where it can bind to circulating IL-6 (106). One study reported significant increases in serum sIL-6R in patients with GD who had active inflammatory TED compared to controls without TED and those with low inflammatory (inactive) TED (105). However localized sIL-6R was not found to be significantly upregulated in TED (99).

Given that IL-6 can be difficult to reliably measure, surrogates of IL-6 pathway activation may serve as alternative biomarkers (107). Currently, studies evaluating surrogates of IL-6 pathway activation (e.g., CRP, neutrophil-to-lymphocyte ratios [NLR]) in TED are limited. CRP production within the liver is induced by IL-6 (108). When compared to healthy controls, CRP was found to be increased in tear-derived exosomes from patients with TED (109). Additionally, NLR is an emerging indicator of systemic inflammation (110) and IL-6 signaling in various diseases (111–113). NLR was found to correlate with CAS, proptosis, and imaging parameters in 87 consecutive patients with TED (114). The same study reported that elevated baseline NLR correlated with reduced clinical outcomes after a median follow-up of 25 months (114). A meta-analysis including 734 subjects also reported significant differences in NLR between TED and control groups (97). Although promising, additional studies are needed to better understand which molecular players involved in IL-6 signaling may serve as appropriate biomarkers for TED.

## 6 Research gaps and future directions

Several knowledge gaps remain as it relates to the epidemiology and pathogenesis, as well as diagnosis and treatment of TED. Currently, the heterogeneous manifestations of TED among different patient demographics are poorly understood and may be due to genetics or other factors (15, 115). Given it’s pivotal role in TED pathogenesis, further investigations into the mechanisms underlying IL-6 signaling are critical. For example, when coupled with TGF-β, IL-6 drives differentiation of Th17 cells; these cells are then maintained and amplified by IL-23 (116). Interestingly, polymorphisms within *IL-23R* are strongly associated with TED; though these findings were not replicated in a subsequent study (34, 117). Additional studies exploring variants within *IL-23R* may provide further insight into the role of Th17 pathway in TED. Beyond *IL-23R*, larger genetic studies may also aid identification of potential causal relationships between *IL-6* polymorphisms and TED.

Additionally, availability and information surrounding clinical biomarkers for TED are extremely limited. Few studies have investigated serum IL-6 levels in patients with TED; conflicting results have also been observed across publications. Studies of other IL-6 pathway markers such as CRP are also limited. This lack of IL-6 pathway biomarkers has hindered clinicians’ ability to properly predict patient responses to IL-6 pathway inhibitors including long-standing options (>10 years) such as tocilizumab. Future studies of potential biomarkers including epigenetic factors, may help improve diagnostic and treatment approaches for TED (3, 118).

Available treatment options such as teprotumumab have surprisingly blurred the line between active and chronic TED. Although data from current publications vary, it is clear that the chronic changes to extraocular muscles and fat once thought to be chronic and permanent can be addressed by treatment interventions such as teprotumumab later in the disease process (26, 52, 72). These new findings also pose challenges of treating a chronic disease already requiring a complex and multimodality approach in the acute onset of presentation. The risks and benefits of each treatment option will need to be evaluated after multiple courses over varying lengths of time. With recent developments in therapies targeting specific components of molecular pathways, it will be important to understand how to optimize the effectiveness of potential therapeutics while mitigating potential short- and long-term side effects due to unintended downstream immunological consequences (71).

In line with the above, there is also a need for well-controlled studies evaluating IL-6 pathway inhibitors for the treatment of TED since data from only one RCT has been published (79). Future studies should aim to assess the tolerability, effectiveness, and QoL impacts of IL-6 pathway inhibitors in patients who are treatment-naïve and those who have received multiple prior modalities of treatment. This will allow for a more comprehensive understanding of the effectiveness and tolerability of IL-6 pathway inhibitors across a broad range of patient types. It will also be important to understand the potential differences in treatment efficacy and tolerability when blocking different components of IL-6R signaling (classical versus trans signaling).

## 7 Conclusion

The IL-6 pathway is increasingly recognized as a potential therapeutic target in TED. IL-6 is significantly upregulated systemically and locally within the orbits of those with TED. However, additional studies examining the genetic associations of IL-6 as well as IL-6 as a biomarker in TED are needed. Given what is generally known about the pleiotropic functions of IL-6 and specifically reported within TED, IL-6 could play roles in autoantibody formation, OF activation and differentiation, and T cell-mediated mechanisms of disease. Clinical studies evaluating treatments that target the IL-6 pathway have reported reductions in proptosis, inflammation (CAS), and thyroid autoantibodies. However, many of these studies were retrospective and in patients who were also steroid-resistant. The utility of IL-6 pathway inhibition for the treatment of TED remains understudied. To date, only one RCT has been completed that evaluates IL-6 pathway blockade in TED; however, several RCTs are currently underway. Development of additional novel therapies will provide healthcare providers with a range of options to tailor treatment to each patient’s specific needs and circumstances, especially over the chronic course of this heterogeneous disease process.
