# Correction: The role of IL-6 in thyroid eye disease: an update on emerging treatments

**DOI:** 10.3389/fopht.2025.1641164

**Published:** 2025-07-16

**Authors:** Jennifer Murdock, John Nguyen, Brady J. Hurtgen, Cathy Andorfer, John Walsh, Andrea Lin, Christopher Tubbs, Kristine Erickson, Kimberly Cockerham

**Affiliations:** ^1^ Jennifer Murdock MD, PLLC, Miami, FL, United States; ^2^ School of Medicine, West Virginia University, Morgantown, WV, United States; ^3^ Tourmaline Bio, Inc., New York, NY, United States; ^4^ Department of Surgery, Sharp Grossmont Hospital for Neuroscience, La Mesa, CA, United States

**Keywords:** IL-6, IL-6R, thyroid eye disease (TED), Graves’ ophthalmopathy, Graves’ orbitopathy, Graves’ disease, thyroid-associated orbitopathy (TAO)

Author Jennifer Murdock was erroneously assigned to affiliation “1Oculofacial Plastic Surgery, Miami, FL, United States, and 2Thrive Health, West Vancouver, BC, Canada” instead of “1Jennifer Murdock MD, PLLC, Miami, FL, United States”. This affiliation has now been updated for author Jennifer Murdock.

*The conflict of interest statement has been corrected to*: AL, BH, CA, CT, JW, and KE are employees of Tourmaline Bio, Inc. JM is a medical director for Thrive Health, which administers IV infusions of teprotumumab, was a consultant for Horizon/Amgen until September 2023, and has a CDA with Tourmaline Bio, Inc. for planning internal consulting and education. JM is also the CEO of Jennifer Murdock MD, PLLC. KC is a consultant and principal investigator for Horizon/Amgen, Viridian, Acelyrin, Genentech/Roche, Immunovant, ArgenX, and Tourmaline Bio, Inc. JN has received research funding from Tourmaline Bio, Inc.

The original article has been updated.

